# W-GUN: Whale Optimization for Energy and Delay-Centric Green Underwater Networks

**DOI:** 10.3390/s20051377

**Published:** 2020-03-03

**Authors:** Rajkumar Singh Rathore, Suman Sangwan, Sukriti Mazumdar, Omprakash Kaiwartya, Kabita Adhikari, Rupak Kharel, Houbing Song

**Affiliations:** 1 Department of Computer Science and Engineering, Deenbandhu Chhotu Ram University of Science and Technology, Murthal (Sonepat), Haryana 131039, India; rajkumar.schcse@dcrustm.org (R.S.R.); suman.cse@dcrustm.org (S.S.); 2 School of Computer and Systems Sciences, Jawaharlal Nehru University, New Delhi 110067, India; sukriti.mazumdar@gmail.com; 3 School of Science and Technology, Nottingham Trent University, Nottingham NG11 8NS, UK; 4 School of Engineering, Newcastle University, Newcastle upon Tyne NE1 7RU, UK; kabita.adhikari@newcastle.ac.uk; 5 Department of Computing and Mathematics, Manchester Metropolitan University, Manchester M15 6BH, UK; r.kharel@mmu.ac.uk; 6 Department of Electrical, Computer, Software, and Systems Engineering, Embry-Riddle Aeronautical University, Daytona Beach, FL 32114, USA; houbing.song@erau.edu

**Keywords:** underwater sensor networks, green computing, whale optimization, sensor networks

## Abstract

Underwater sensor networks (UWSNs) have witnessed significant R&D attention in both academia and industry due to their growing application domains, such as border security, freight via sea or river, natural petroleum production and the fishing industry. Considering the deep underwater-oriented access constraints, energy-centric communication for the lifetime maximization of tiny sensor nodes in UWSNs is one of the key research themes in this domain. Existing literature on green UWSNs are majorly adapted from the existing techniques in traditional wireless sensor network relying on geolocation and the quality of service-centric underwater relay node selection, without paying much attention to the dynamic underwater network environments. To this end, this paper presents an adapted whale and wolf optimization-based energy and delay-centric green underwater networking framework (W-GUN). It focuses on exploiting dynamic underwater network characteristics by effectively utilizing underwater whale-centric optimization in relay node selection. Firstly, an underwater relay node optimization model is mathematically derived, focusing on underwater whale dynamics for incorporating realistic underwater characteristics in networking. Secondly, the optimization model is used to develop an adapted whale and grey wolf optimization algorithm for selecting optimal and stable relay nodes for centric underwater communication paths. Thirdly, a complete workflow of the W-GUN framework is presented with an optimization flowchart. The comparative performance evaluation attests to the benefits of the proposed framework and is compared to state-of-the-art techniques considering various metrics related to underwater network environments.

## 1. Introduction

The widely growing application domains for underwater sensor networks (UWSNs) have attracted potential attention in R&D from the Internet of Things (IoT)-oriented industries and academia [1,2,3]. The growing domains include border security-centric military applications [4,5], energy and cost-centric water-based transport applications [6,7], oil, and natural gas production applications [8,9], and developing fishing-centric industries [10,11]. In underwater networking, tiny sensor nodes are deployed underwater, as well as on the upper surface layer for monitoring the specific underwater area [12]. These underwater nodes communicate with the surface nodes, acting as access points or cluster heads for reaching the sink node of the network, which accumulates the information and communicates with the cloud-enabled computing resources [13]. Underwater networking is significantly challenging compared to traditional wireless networking due to the dynamic self-mobility of the medium of communication and constraints in signal propagation in the underwater environment [14,15,16]. In this constrained networking environment, the underwater network deployment-oriented challenges further complicate scientific investigations towards the development of an energy-centric green underwater network for various application domains [17,18,19].

Towards enabling green underwater networking, several service and geolocation-centric techniques of varying quality have been suggested [20,21]. A heuristic approach has been suggested in underwater networking for solving the surface gateway deployment optimization problem, focusing on the quality of service [22]. In particular, a heuristic solution has been explored for the optimal deployment of surface nodes as access gateways for underwater networking. However, the surface gateway deployment optimization lacks coordination with underwater node level multi-hop communication. To support service-centric underwater networking, an optimal underwater node deployment architecture is explored for a 3D underwater network environment [23]. A scientific, mathematical model is designed for assessing the optimality of the deployment architecture. However, the deployment architecture did not integrate with the dynamic self-mobility of underwater nodes in the network environment. The 3D deployment architecture has been further improved by considering static, self-adjusting, and mobility-supported deployments [24]. However, the coordination between static and dynamic nodes during communication is lacking in these three types of deployment architectures. To support these deployment approaches, a linear programming-centric approach has been suggested for selecting underwater relay nodes focusing on a longer network lifetime [25].

In a similar work, node-level energy harvesting capability has been used as a parameter in relay node selection for a longer network lifetime [26]. However, the impact of self-mobility of underwater nodes due to underwater flow is not considered in both approaches, including deployment-centric network lifetime optimization and harvested energy level-centric network lifetime optimization. To improve network lifetime optimization, a geolocation vector-based forwarding strategy has been explored [27]. It has focused on location-centric relay node selection to reduce energy consumption in underwater communication. The geolocation-centric underwater relay node selection has been improved by utilizing hop-by-hop forwarding prioritization for sparse underwater networking [28]. The pure geolocation-centric relay node selection faces the void area issue in underwater networking. To address the issue in geolocation-centric relay node selection, void avoidance approach has been investigated, utilizing the quality of service-oriented underwater backtracking [29]. The aforementioned underwater relay node optimization techniques majorly rely on either geolocation-centric node selection or the quality of service-centric node selection, without considering the dynamic self-mobility of the medium of communication in underwater environments, such as in whale optimization [30].

In this context, this paper proposes an adapted whale optimization algorithm-based energy and a delay-centric green UWSNs framework (W-GUN). It focuses on exploiting dynamic underwater network characteristics by effectively utilizing underwater whale-centric optimization in relay node selection. The significant insights and offerings of this paper can be listed as follows.

- Firstly, an underwater relay node optimization model is mathematically derived, focusing on underwater whale dynamics for incorporating realistic underwater characteristics;
- Secondly, the optimization model is used to develop an adapted whale and grey wolf optimization algorithm for selecting minimal energy consumption and stable relay node-centric underwater communication paths;
- Thirdly, a complete workflow of the green underwater networking framework W-GUN, is presented with an optimization flowchart;
- Finally, a comparative performance evaluation of the prosed framework W-GUN, has been carried out considering the state-of-the-art techniques in the literature regarding underwater networks.

The rest of the paper is organized as follows: in Section 2, related works on green computing-centric underwater networks are critically reviewed, considering strengths and weaknesses. Section 3 presents the detail of the proposed green underwater networking-centric framework W-GUN. In Section 4, the experimental setting and result analysis are discussed. Our conclusions and possibilities for future work are been presented in Section 5.

## 2. Related Works

Ibrahim et al. use a heuristic approach to solving the surface gateway deployment optimization problem. The performance of UWSNs can be increased by deploying various surface-level gateways (i.e., sink for the UWSNs). In addition, this approach can mitigate the high propagation delay in acoustic communications. The position of gateways plays a crucial role in maximizing the benefit [22]. Furthermore, Pompili et al. proposed the enhanced deployment schemes for two-dimensional as well as for three-dimensional architectures of communication in UWSNs. They also gave a mathematical analysis for both scenarios. The proposed scheme helps in achieving the goal of using the least number of sensors for efficient sensing and communication tasks. They discussed the robustness of the sensor network in the particular scenario of node failures. They also gave an approximate count of the number of redundant sensor nodes to be deployed to compensate in case of node failures [23]. Moreover, Liu et al. presented an efficient algorithm for node placement in UWSNs. The aim is to enhance the coverage and reduce the average end-to-end delay. They use the new tracking scheme to forecast the sensor node’s positions. The deployment is carried out by using two factors, such as the current location of sensor nodes as well as predicted locations of sensor nodes [24]. Su et al. have proposed a unique approach for selecting the relay nodes in UWSNs. They use the linear programming approach for the relay node selection with the principle aim of enhancing the network lifetime. They also implemented a routing metric that considers both the transmitting energy and the residual energy [25]. Additionally, Khan et al. presented a scheme for relay node selection in UWSNs based on harvested energy levels. For the selection of the correct signal at the destination, they used a fixed combined ratio. Furthermore, they used an amplified forwarding scheme for data forwarding. In addition, they used the piezoelectric effect-based harvesting scheme to increase the efficiency of sensors in UWSNs [26]. 

Xie et al. have proposed an efficient routing scheme named vector-based forwarding (VBF). It is a robust, scalable, and energy-efficient geographic routing. This scheme is also called a position-based technique. In this scheme, there is no need for detailed state information. The scheme is called a robust scheme because it is immune to packet loss and node failure. The data transmission mechanism consists of a virtual pipe. In the case of dense deployment, energy consumption is increased. The sparse networks have a low packet delivery ratio (PDR), but, in the case of dense networks, it increases. This scheme has a lower end-to-end delay. The end-to-end delay is minimum in a dense environment. However, this scheme produces communication overheads [27]. Towards enhancing the vector-based forwarding, Nicolaou et al. have given a location-based routing technique known as hop-by-hop vector-based forwarding (HH-VBF). Each forwarder has a separate routing vector in the network. The separate routing vector for each forwarder brings several advantages and achieves a high efficiency for sparse networks. This scheme has a higher packet delivery ratio in sparse networks. This scheme has unique the characteristic of recognizing the routes, particularly when nodes are extremely scattered in the network. In this scheme, if node density increases, then, as a consequence, end-to-end delay, as well as energy consumption, also increases [28]. In addition, UWSNs are blighted by one of the most prominent issues, known as the routing under the void scenario. 

Xie et al. have proposed a unique approach to solving this problem. This approach is known as vector-based void avoidance (VBVA). This approach has two parts, vector-shift and back-pressure. This scheme does not require prior knowledge of network topology. This technique is a geographic-based routing. This approach enhances the robustness of the network [29]. Next, Han et al. classified the node deployment schemes for UWSNs into three major categories, static or fixed scheme of deployment, the self-adjusting scheme of deployment, and movement-supported deployment. In the static or fixed scheme of deployment, sensors have fixed or static positions. There are two types of fixed schemes of deployment—random deployment and regular deployment. In the self-adjusting scheme, the depths of sensor nodes are adjusted automatically after initial deployment in order to achieve the specific requirements. Furthermore, there are two types of self-adjusting schemes of deployment—such as uniform self-adjusting deployment and nonuniform self-adjusting deployment. In a movement-supported deployment scheme, sensor nodes cooperate with other sensors to carry out sensing and monitoring tasks [31]. 

Furthermore, Khan et al. have proposed an optimal scheme for relay selection in UWSNs. In this scheme, they consider two factors, the depth and location of the sensor nodes, for selecting the relay nodes. The unique characteristic of the proposed scheme is the elimination of the synchronization requirement among the source node (SN), relay nodes (RNs), and destination nodes. The second unique feature is overcoming the packet drop issues [32]. Next, Feng et al. have proposed an algorithm consisting of two parts—the establishment of links and transmission of data. The algorithms find out the neighboring nodes at the appropriate ranges. In addition, the proposed algorithm selects the relay nodes based on the depth of the neighbors. For balancing the energy in the network and consequently increasing the network lifetime, the communication links were modified [33]. Moreover, Faheem et al. proposed a routing protocol that uses three basic schemes, the detection of the channel, then the assignment of the channel and packet-forwarding mechanisms. The channel detection scheme had a high channel detection probability and the least false alarms [34]. Next, Wei et al. have presented an optimal strategy for routing in UWSNs. This scheme initially considers residual energy and localization information. This routing mechanism collects information about the position for energy saving with vector-based forwarding (ES-VBF) [35].

The aforementioned underwater relay node optimization techniques rely majorly on either geolocation-centric node selection or the quality of service-centric node selection, without considering the dynamic self-mobility of the medium of communication in underwater environments. To this end, the objective of the proposed framework is to reduce energy consumption in the network by adapting underwater whale characteristics for the optimization of the overall performance of underwater networking.

## 3. Green Underwater Sensor Network Framework using Adapted Whale and Wolf Optimization

In this section, the proposed framework for the green underwater network using whale and wolf optimizations (W-GUN) is presented. In UWSNs, researchers are currently aiming to increase network lifetime and improve the data delivery rate, considering constrained underwater network scenarios. For this, the data dissemination path should consider natural underwater characteristics along with the shortest path possible. It will lower the energy consumption and also packet delay in the underwater scenario. However, the best deployment of underwater relay nodes optimizes each network resource and routing performance. Therefore, optimizing the number of underwater relay nodes and the deployment strategy of relay nodes considering underwater scenarios has been an essential downside to green communication in underwater networks.

In order to solve the above mentioned problem, this paper presents a novel approach for the optimization of underwater relay nodes prioritization using an adapted whale and wolf optimization algorithm. The adapted optimization algorithm has been developed to realize the W-GUN framework by incorporating moving underwater whale characteristics in underwater data dissemination. Here, the optimal best relay nodes are obtained simultaneously from the algorithms, including whale optimization and wolf optimization, and the final relay node decision is taken to select the best among them during each iteration. At the completion of every iteration, the best underwater relay node solution will be given to both algorithms to generate a better solution for the underwater relay node than the previous one, and the same cycle is repeated. Here, the deployment of underwater sensor nodes comes in the form of normal random distribution at the initial underwater network establishment stage.

By utilizing the proposed adapted optimization algorithms in W-GUN, the minimum number of underwater relay nodes required is specifically determined once the network parameters are provided, such as the variety of source nodes, the communication range, and amount of routing paths. Once the underwater relay nodes are optimally determined, the average path length and, therefore, the range of control packets were significantly reduced, which may doubtless minimize energy consumption and packet delay and increase network lifetime, and packet delivery ratio. Moreover, the duty cycle of underwater relay nodes is adjusted adaptively using the modified echo state network (MESN).

### 3.1. Underwater Relay Node Optimization Model

Here, as the adapted whale and wolf optimization algorithm is used for picking the optimal relay set required for continuous data packet transmission in W-GUN. In our proposed framework W-GUN, the traditional whale optimization is adapted for green underwater networking. The exploitation and exploration of whale optimization is further improved via wolf optimization. This adaptation works towards harnessing the benefits of underwater whale movement characteristics in reducing energy consumption and delay. The adaptation of whale movement characteristics is crucial for underwater communication environments. The aim of the objective function of the optimization scheme is to avail the maximum number of underwater forwarding routes as possible reachable paths with a minimum number of underwater relay nodes. So, the position of the underwater relay nodes is varied and checked for optimal deployment, so as to reduce the number of underwater relay nodes (RNs) needed for deployment. Therefore, the objective function is adapted for underwater scenarios, as expressed in Equation (1).

(1) $$max\left(P_{n}\right)=min(R_{n}^{b},\sum_{j=1}^{N}R_{n}^{j})$$

where $max\left(P_{n}\right)$ represents the maximum number of underwater forwarding paths that can be formed between the base station and all sender sensor nodes, $R_{n}^{b}$ represents the number of underwater relay nodes nearby to the base station; $R_{n}^{j}$ the number of underwater relay nodes close to *j*th sensor node and j={1,2,…N} denotes the number of underwater sensor nodes. To achieve the above objective, the two major constraints that must be satisfied are as given below in Equations (2) and (3):

(2) $$R_{n}^{j}\geq P_{n}^{j}$$

(3) $$R_{n}^{b}\geq \sum_{j=1}^{N}R_{n}^{j}\geq (\sum_{j=1}^{N}P_{n}^{j}=P_{n})$$

where $P_{n}^{j}$ denotes the number of underwater forwarding paths between the base station and *j*th underwater relay sensor nodes. During each iteration, based on the maximum number of underwater forwarding paths obtained for different solutions, the set of underwater relay nodes (i.e., the finest solution) and their position is selected. The base of the objective function of the adapted whale and wolf algorithm is discussed in detail, with related mathematical derivations, in the following sections.

#### 3.1.1. Adapted Whale Optimization for Underwater Networks

The whale optimization algorithm (WOA) is influenced by the natural characteristics of the underwater movement of whales [30]. The technical optimization steps involved in WOA are given in the following description, which can be divided into two major phases, namely exploitation and exploration phases. In the exploitation phase, the encircling prey and spiral position updating are performed, and searching for prey is implemented in the exploration phase. The mathematical modeling of these operations is carried out as follows:

• Encircling prey

Traditionally, whales encircle the prey once they discover its location or position. Similarly, the position of the optimal next-hop sensor nodes is identified in the underwater network environments. In the proposed algorithm, the present leading candidate solutions, i.e., the positions of the direct neighbor nodes, are the target prey near the optimal solution. Subsequently, the other search agents attempt to change their position towards the best search agents. The encircling prey can be mathematically represented as given in Equation (4):

(4) $$\vec{W}=\left|\vec{G}\cdot {\vec{M}}^{*}\left(k\right)-\vec{M}\left(k\right)\right|$$

(5) $$\vec{M}\left(k+1\right)={\vec{M}}^{*}\left(k\right)-\vec{X}\cdot \vec{W}$$

where ‘k’ represents a current iteration, $\vec{X}$, $\vec{G}$ represent coefficient vectors, ${\vec{M}}^{*}\left(k\right)$ demonstrate the previous best solution or position of the previous best node, $\vec{M}\left(k+1\right)$ depicts the current best state or the position of the current best node for next-hop forwarding in the neighborhood. Furthermore, the coefficient vectors $\vec{X}$, $\vec{G}$ can be calculated as expressed in Equations (6) and (7):

(6) $$\vec{X}=2\vec{x}\cdot \vec{J}-\vec{x}$$

(7) $$\vec{G}=2\cdot \vec{J}$$

where $\vec{x}$ reduces from 2 to 0, $\vec{J}\in \left[0,1\right]$ also results in the reduced range of $\vec{X}$. The new position of the current best forwarding node can be determined anywhere between the previous best position of the node and the encircling preposition.

• Exploitation phase

There are two mechanisms presented to carry out the exploitation phase in the whale-based search space discovery:

*1. Shrinking encircling mechanism*

This mechanism is basically represented in Equation (6) where the value of the unit coefficient vector $\vec{x}$ is reduced from 2 to 0 with $\vec{J}\in \left[0,1\right]$.

*2. Spiral updating position*

Traditionally, once the distance between the encircling prey and the whales is calculated, a spiral equation is derived between the position of the prey and the whales to imitate the helix-shaped movement of whales. Here, the positions of the current forwarder node and the direct neighbor nodes are shown in Equation (8):

(8) $$\vec{M}\left(k+1\right)={\vec{W}}_{dist}.{exp}^{{log}_{spiral}s}\cdot cos\left(2\prod s\right)+{\vec{M}}^{*}\left(k\right)$$

where ${\vec{W}}_{dist}=\left|{\vec{M}}_{p}^{*}\left(k\right)-\vec{M}\left(k\right)\right|$ means the distance between pth whale and prey (i.e., the best solution attained up until now), s takes value from [−1,1] and $log_{spiral}$ signifies the logarithmic spiral shape.

Here, we want to highlight that the ‘exploitation phase’ of the whale optimization process can be executed by either ‘shrinking encircling’ or ‘spiral updating’. These operations basically represent humpback whales’ swimming characteristics around the prey within a shrinking circle and along a spiral-shaped path, simultaneously. To model this simultaneous behavior, the selection of the threshold value plays a significant role, where we assume that there is a 0.5 probability of choosing between either the shrinking encircling mechanism or the spiral model for the next future position of the whales during optimization. This is assumed in order to give a fair amount of randomization to the whale optimization process for underwater networking environments. This exploitation phase can be expressed as given in Equation (9):

(9) $$\vec{M}\left(k+1\right)=\{\begin{array}{c} {\vec{M}}^{*}\left(k\right)-\vec{X}\cdot \vec{W},if\;Q<0.5\\ {\vec{W}}_{dist}.{exp}^{{log}_{spiral}s}\cdot cos\left(2\prod s\right)+{\vec{M}}^{*}\left(k\right),ifQ\geq 0.5 \end{array}$$

where Q∈[0,1] is a random number to help with the selection of the two aforementioned mechanisms in the underwater sensor node selection for energy- and delay-centric next-hop node identification or, in other words, search space exploitation.

• Exploration phase

Traditionally, it is the search phase where whales use random search to discover their prey based on the how nearby they are. Here, the search for the next-hop is carried out using the nearby positions of the direct neighbor nodes, which are candidate forwarders. The exploration uses $\vec{X}$ vector with random values that are greater or less than one. In addition, a random search agent is considered, rather than the best search agent for addressing the optimal local problem in the exploration phase. This search procedure can be mathematically expressed as given in Equation (10):

(10) $$\vec{W}=\left|\vec{G}\cdot {\vec{M}}^{rand}-\vec{M}\right|$$

(11) $$\vec{M}\left(k+1\right)={\vec{M}}^{rand}-\vec{X}\cdot \vec{W}$$

where ${\vec{M}}^{rand}$ is a current population random position vector, and $\vec{G}$ and $\vec{X}$ are coefficient vectors, as described in previous equations. Now, grey wolf optimization (GWO) is discussed, which enables the whale optimization for underwater node searching.

#### 3.1.2. Adapted Grey Wolf Optimization for Underwater Networks

Towards effectively balancing the exploitation and exploration phases for better convergence in next-hop searching, wolf optimization is used to enable the proposed framework W-GUN [36]. It is also a swarm intelligence technique. In traditional GWO, tracking is directed by the alpha, beta and delta wolves as follows:

- The alpha wolves (α): the most important wolves. They encompass a responsibility to make a decision. Using this setting, we focus on the energy-centric nodes for optimization;
- The beta wolves (β): they comprise the second tier of wolves, subsequent to the alphas. The standard responsibility of beta wolves is to aid and encourage alpha selection. Using this setting, we focus on delay-centric nodes for optimization;
- The delta wolves (δ): they comprise the third tier of wolves. Using this setting, both energy and delay are considered as the overall optimization goal.

In W-GUN, the most important aspiration is to encircle a prey by guidance through *α*, *β* and *δ*, which can be systematically established as given in Equation (12):

(12) M(k+1)=M(k)−C⋅P

(13) $$P=\left|V\cdot M_{l}\left(k\right)-M\left(k\right)\right|$$

where P is the search accelerator parameter, M represents the grey wolf position, $M_{l}$ is the prey position, C, V are the coefficient vectors, and the number of iterations is defined by ‘k’. The coefficient vectors C and V can be obtained by the equation below:

(14) $$C=2c\cdot w_{1}-c$$

(15) $$V=2\cdot w_{2}$$

where ‘c’ will be linearly decreased from 2 to 0 and $w_{1}$ and $w_{2}$ are the random vectors from [0, 1]. The parameter ‘c’ is updated in every iteration within the range from 2 to 0, according to the below Equation (16):

(16) $$c=2-k\left(\frac{2}{K}\right)$$

where ‘K’ denotes the total number of iterations allowed. It is assumed that the large number of possible locations of prey can be discovered through the alpha, beta, and delta solutions; the updated procedure of the whales positions, based on the first three best solutions, can be obtained as shown below:

(17) $$M_{1}=M_{\alpha }\left(k\right)-C_{1}\cdot P_{\alpha }$$

(18) $$M_{2}=M_{\beta }\left(k\right)-C_{2}\cdot P_{\beta }$$

(19) $$M_{3}=M_{\delta }\left(k\right)-C_{3}\cdot P_{\delta }$$

where the component values $P_{\alpha }$, $P_{\beta }$ and $P_{\delta }$ are calculated as follows:

(20) $$P_{\alpha }=\left|V_{1}\cdot M_{\alpha }-M\right|$$

(21) $$P_{\beta }=\left|V_{2}\cdot M_{\beta }-M\right|$$

(22) $$P_{\delta }=\left|V_{3}\cdot M_{\delta }-M\right|.$$

Based on the above Equations (17)–(19), the solution for the next iteration will be obtained as follows:

(23) $$M\left(k+1\right)=\frac{\left(M_{1}+M_{2}+M_{3}\right)}{3}$$

**Figure 1:**
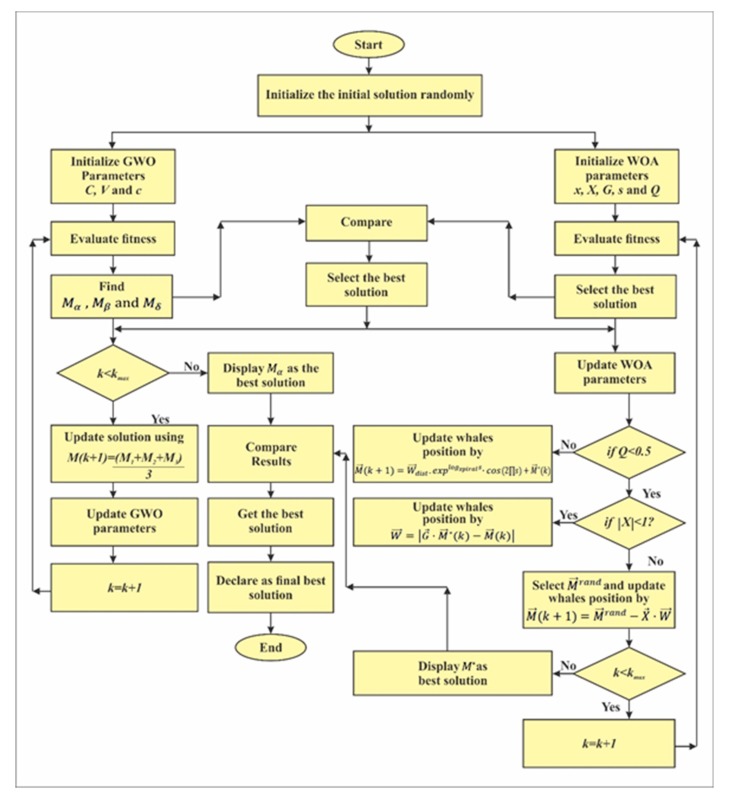
Flowchart of the proposed adapted whale optimization for the green underwater network (W-GUN).

The process of updating the whales positions takes place continuously until the maximum iteration is achieved. A complete procedure of the proposed framework W-GUN, is presented below as an algorithm and flowchart in Figure 1.

### 3.2. The Pseudo-Code for the Proposed W-GUN Framework

In Algorithm 1, a few of the major operations of the optimization model are detailed. In steps one and two, initialization of the optimization model is performed, which is basically a constant time operation depending on the population size $M_{x}$. In step two, the fitness function for the optimization model is calculated using Equation (1). In step five, whale optimization is considered for better underwater relay node selection. The exploration and exploitation of whale optimization is enhanced in step six via the wolf optimization method. In steps 10–13, we prioritize the three best solutions as the most recent solutions at each optimization iteration. In steps 17–31, the whale optimization-based iterative search is performed for selecting underwater relay nodes. In steps 35–38, the wolf optimization-based search is performed for enhancing the exploitation and exploration of the whale approach. In steps 40–44, the three optimal relay node solutions are prioritized for each particular iteration.

**Algorithm 1** W-GUN: Whale-centric Optimization for Green Underwater Networks
*1*.***Initialize** the population,*$M_{x}\left(x=1,2,\ldots ,y\right)$,
*//* $M_{x}$ *represents the set of random solutions based on the size and position of relay nodes*
*2*.
***Initialize** WOA parameters(x, X, G, Q and s) and GWO parameters(C, V and c)*
*3*.
*Calculate the **fitness**($FF=max\left(P_{n}\right)$) of each search agent using Equation (1)*

*// Fitness represents the maximum number of paths covered with minimal relay nodes*
*4*.
*Selection based on the adapted search algorithm*
*5*.
***For** WOA-based search*
*6*.
*Find the best solution* $M^{*}$
*7*.
***For** GWO-based search*
*8*.
*Do*
*9*.
*Separate the solutions based on the fitness*
*10*.
$M_{\alpha }$ *= the first best search solution*
*11*.
$M_{\beta }$ *= the second-best search solution*
*12*.
$M_{\delta }$ *= the third best search solution*
*13*.Compare $M^{*}$ *and* $M_{\alpha }$
*//Select the best solution among* $M^{*}$ *and* $M_{\alpha }$ *pass* M(final_best) *to WOA- and GWO-based updating procedures*
*14*.***While*** (k *< maximum number of iterations)**15*. *Do update based on WOA and GWO**16*. *// WOA-based update**17*. ***For** each search agent**18*.  *Update* $\vec{x},\vec{X},\vec{G}$, Q *and* s*19*.  ***if1***(Q<0.5)*20*.   ***if2***($\left|\vec{X}\right|<1$)*21*.    *Update the position of the current search agent by,**22*.    $\vec{W}=\left|\vec{G}\cdot {\vec{M}}^{*}\left(k\right)-\vec{M}\left(k\right)\right|$*23*.    ***else if2***($\left|\vec{X}\right|\geq 1$)*24*.    *Select a random search agent* (${\vec{M}}^{rand}$)*25*.    *Update the position of the current search agent by,**26*.    $\vec{M}\left(k+1\right)={\vec{M}}^{rand}-\vec{X}\cdot \vec{W}$*27*.   ***end if2****28*.  ***else if1***(Q≥0.5)*29*.  *Update the position of the current search by,**30*.  $\vec{M}\left(k+1\right)={\vec{W}}_{dist}.{exp}^{{log}_{spiral}s}\cdot cos\left(2\prod s\right)+{\vec{M}}^{*}\left(k\right)$*31*.  *(where* ${\vec{W}}_{dist}=\left|{\vec{M}}_{p}^{*}\left(k\right)-\vec{M}\left(k\right)\right|$ *It means the distance between* $p^{th}$ *whale and prey* (i.e., *the best solution attained till now);* s∈[−1,1] *and* $log_{spiral}$ *is the constant for defining the shape of the logarithmic spiral)**32*.  ***end if1****33*. ***end for****34*. ***Return*** $M^{*}$ *//GWO-based update**35*. ***For** each search solution**36*.  *Update the current search agent**37*.  $M\left(k+1\right)=\frac{\left(M_{1}+M_{2}+M_{3}\right)}{3}$*38*.  *where* $M_{1}=M_{\alpha }\left(k\right)-C_{1}\cdot P_{\alpha };M_{2}=M_{\beta }\left(k\right)-C_{2}\cdot P_{\beta };M_{3}=M_{\delta }\left(k\right)-C_{3}\cdot P_{\delta };$           $P_{\alpha }=\left|V_{1}\cdot M_{\alpha }-M\right|;P_{\beta }=\left|V_{2}\cdot M_{\beta }-M\right|;P_{\delta }=\left|V_{3}\cdot M_{\delta }-M\right|;$*39*. ***End for***
*40*. *Check if any search agent goes beyond the search space and adjust it**41*. *Calculate the fitness of each search agent (i.e., relay set) obtained through both search algorithms**42*. *Compare and update* M(final_best) if there is a better solution*43*. *Store the best solution attained so far**44*. k=k+1*45*.
***end while***
*46*.
***return*** M(final_best)
*47*.
*Stop*



### 3.3. The Complete Working Structure of the Adapted Optimization Framework (W-GUN)

**Figure 2:**
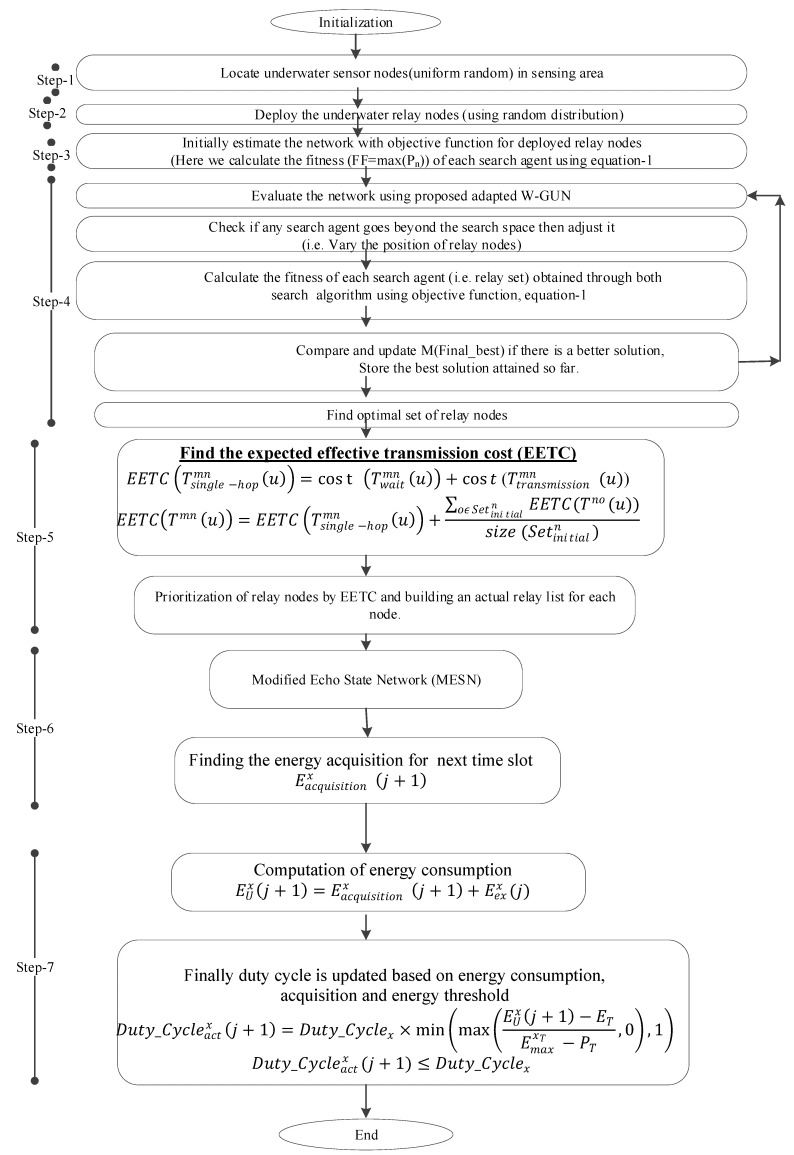
The working structure of proposed adapted relay node optimization framework W-GUN.

The working structure of the adapted whale optimization-based relay node optimization for the W-GUN framework with a dynamic duty cycle is given in Figure 2.

Next, we will discussed the working of the proposed underwater framework W-GUN, in detail as per Figure 2 (above). In the first step, the underwater sensor nodes that were deployed in a uniform random fashion are located in the underwater network sensing area. In the second step, underwater relay nodes deployed in a normal random distribution fashion are identified. In the third step, the initial estimation of the network is performed with the objective function of the deployed underwater relay nodes. Thus, this step initially calculates the fitness of each search agent. In the fourth step, the adapted underwater optimization framework W-GUN, is utilized to find the optimal set of underwater relay nodes from the direct neighbor nodes. A flowchart of the adapted whale and wolf optimization framework is given in Figure 1. The adapted underwater optimization has been designed by integrating the whale and wolf optimization techniques. Here, the optimal best relay node solutions are obtained simultaneously from both algorithms, and the final decision is taken to select the best relay node among them during each optimization iteration. At the completion of every underwater relay node optimization iteration, the best underwater relay node solution will be given to both optimization algorithms to generate solutions that are better than the previous underwater relay node solutions, then the same cycle is repeated. Thus, the output of the fourth step is an optimal set of underwater relay nodes to be used as the next-hop forwarder.

After finding the optimal set of underwater relay nodes, their current duty cycle is calculated in the fifth step. Now, the underwater relay set for each node is identified, and all relay nodes become active in the underwater network path. In addition, in the fifth step, the expected effective transmission cost (EETC) is calculated, and relay nodes are prioritized accordingly [37,38]. Therefore, the actual underwater relay node list for each node is discovered in this step. For each sender node m, the underwater relay nodes n are identified. The EETC of the underwater single-hop data forwarding, $EETC\left(T_{single-hop}^{mn}\left(u\right)\right)$, at particular slot u, is derived from the sum of the underwater transmission cost and the waiting cost, as expressed in Equation (24):

(24) $$EETC\left(T_{single-hop}^{mn}\left(u\right)\right)=cost\left(T_{wait}^{mn}\left(u\right)\right)+cost\left(T_{transmission}^{mn}\left(u\right)\right)$$

where $T_{wait}^{mn}$ is the waiting time interval and $T_{transmission}^{mn}$ represents a transmission time interval. The expected time interval between receiving a packet and beginning to send that packet to other nodes is considered to be the waiting cost. The underwater multi-hop data forwarding uses the single-hop EETC and an average of subsequent underwater relay nodes. This can be calculated as given in Equation (25):

(25) $$EETC\left(T^{mn}\left(u\right)\right)=EETC\left(T_{single-hop}^{mn}\left(u\right)\right)+\frac{\sum_{o\in Set_{initial}^{n}}EETC\left(T^{no}\left(u\right)\right)}{size\left(Set_{initial}^{n}\right)}$$

where $size\left(Set_{initial}^{n}\right)$ is the size of the relay set, $EETC\left(T_{single-hop}^{mn}\left(u\right)\right)$ is the expected effective transmission cost for a single hop of the receiver node n in the relay node-set $Set_{initial}^{n}$. Moreover, $\frac{\sum_{o\in Set_{initial}^{n}}EETC\left(T^{no}\left(u\right)\right)}{size\left(Set_{initial}^{n}\right)}$ is the average dynamic transmission cost of the relay node-set $size\left(Set_{initial}^{n}\right)$.

In step six, underwater node energy acquisition of the next slot is predicted by using a modified eco state network (MESN) model [39]. Here, for (*j* + 1) time slot, energy acquisition is $E_{acquisition}^{x}\left(j+1\right).$ Finally, in step seven, underwater node energy consumption is calculated, and the duty cycle is adjusted accordingly based on energy consumption, the energy acquisition of the next slot and the energy threshold of the underwater network environments.

In the underwater network, the energy utilization of ${\left(j+1\right)}^{th}$ the time slot is nothing but the sum of the energy acquisition of the next time slot and the excess energy of the current time slot, which can be referred to as given in Equation (26):

(26) $$E_{U}^{x}\left(j+1\right)=E_{acquisition}^{x}\left(j+1\right)+E_{ex}^{x}\left(j\right)$$

where $E_{acquisition}^{x}\left(j+1\right)$ denotes the underwater node energy acquisition of next time slot, $E_{ex}^{x}\left(j\right)$ represents the excess energy in the current time slot. By utilizing Equation (2), the dynamic duty cycle in underwater networking as calculated as given in Equation (27):

(27) $$Duty\_Cycle_{act}^{x}\left(j+1\right)=Duty\_Cycle_{x}\times min\left(max\left(\frac{E_{U}^{x}\left(j+1\right)-E_{T}}{E_{max}^{x_{T}}-E_{T}},0\right),1\right)$$

where $Duty\_Cycle_{act}^{x}\left(j+1\right)$ represent the active time at slot (j+1) of the underwater relay node, $Duty\_Cycle_{x}$ is the slot length, $E_{U}^{x}\left(j+1\right)$ refers to the energy utilization of ${\left(j+1\right)}^{th}$ time slot for the underwater relay node, $E_{max}^{x_{T}}$ represent maximum consumption of energy for a slot, and $E_{T}$ represent the energy threshold in underwater communication network environments. The active-duty cycle constraint is referred to as, $Duty\_Cycle_{act}^{x}\left(j+1\right)\leq Duty\_Cycle_{x}$. In addition, if $Duty\_Cycle_{act}^{x}\left(j+1\right)=0$, this represents that the underwater node is entirely inactive for the ${\left(j+1\right)}^{th}$ time slot.

## 4. Results and Discussion

### 4.1. Experimental Settings

In this section, a brief description of the experimental settings is provided, which were used to set up Aqua-Sim-enabled network simulator (ns2) environments for evaluating the performance of the proposed underwater relay node optimization framework W-GUN. The underwater simulation environment had utilized acoustic channels at Medium Access Control (MAC) and physical layers. Towards benchmarking a centric comparative experimental analysis, recent techniques in underwater relay node optimization were considered, including VBF [27], HH-VBF [28], VBVA [29] and ES-VBF [35]. Both VBF and HH-VBF possess the qualities of service-oriented modeling without considering underwater network characteristics in the modeling. VBVA and VBF both focused on void avoidance without topology knowledge using the geographic locations of relay nodes. Here, the significant impact of underwater characteristics on relay node locations is not considered. The adapted optimization framework W-GUN for underwater relay node selection with a dynamic duty cycle is implemented under realistic 3D underwater network environments of size 1000m×1000m×1000m. Here, the underwater sensor nodes vary from 100 to 500 in the network region, which is quite a realistic assumption for scalability. Each sensor node in the region is charged with an initial energy of 100 j. For sending a single packet of size 512 bytes per node, the transmission energy per node is considered 2w, and receiving energy per node is considered to be 0.75w, given that the ideal node needs 10 mw of energy. The transmission range of each underwater sensor node is 150 m, and the movement of underwater nodes in the horizontal dimension is considered to be 0–3 m/s. There is the consideration of an 100 s beaconing gape in underwater networking communication. Due to the longer propagation delay in underwater networking environments, the handling packet collision is significant. The packets colliding in one node may not collide with other nodes or arrive in different sequential orders in underwater environments. This is handballed effectively in our Aqua-Sim-based simulations where every node maintains a local copy of incoming packets and collision of packets are identified using the difference in received power levels locally. Therefore, the effect of collision only remains on local copies of a node and does not impact the copies of other nodes. This is the way in which collisions are handled locally at each node in our simulation experiments. For controlling the constrains of underwater physical layer implementation, we are essentially setting the exposed interface values of the simulator. For example, as attenuation model setting, spreading factor was considered to be two, along with the absorption coefficient calculated following Thorp’s equation with 1500 m/s propagation speed. An average of 50 simulation runs are averaged to get the results, and overall simulation time considered to be 1500 s. A confidence interval of 98% was considered for generating the results. The framework is implemented in the C++ environment in the simulator.

### 4.2. Analysis of Results

**Figure 3:**
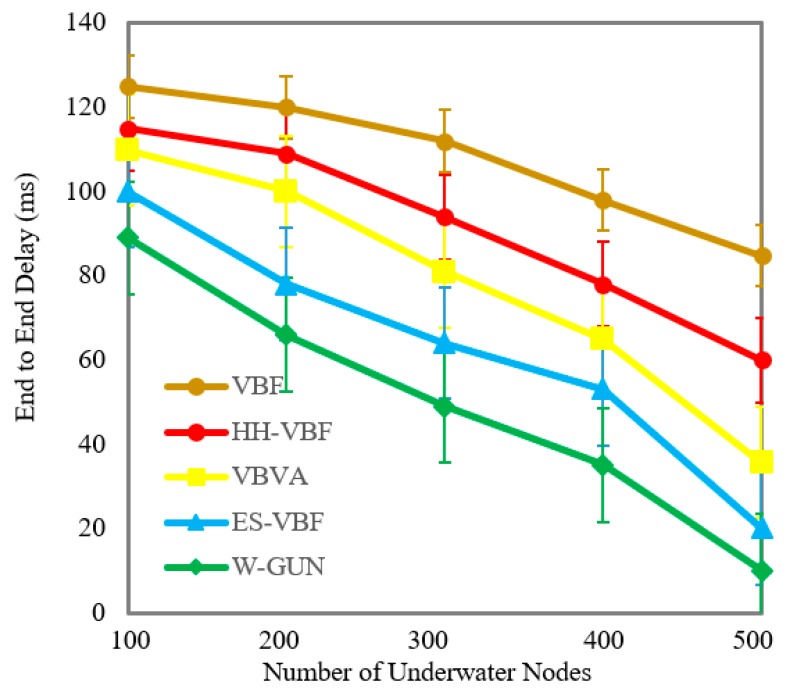
End-to-end delay versus the number of underwater nodes.

This section covers the comparative performance analysis part of the proposed approach with the existing methods. The analysis of the end-to-end delay versus the number of underwater sensor nodes is given in Figure 3. Here, the number of underwater sensor nodes is varied as 100, 200, 300, 400, and 500. It shows that the delay is considerably less for the proposed underwater relay optimization method W-GUN than the state-of-the-art techniques. It is evident that the optimal selection of underwater relay nodes enhances the performance of the overall network, resulting in reduced delay. For a lower delay, the underwater routing path should be as optimal as possible. The aim of the proposed optimization method is to select the maximal number of underwater routes with a minimal number of underwater relay nodes. Therefore, the route consists of the minimum number of underwater relay nodes in our proposed approach. Thus, the delay is considerably minimal compared with existing schemes in the underwater literature.

**Figure 4:**
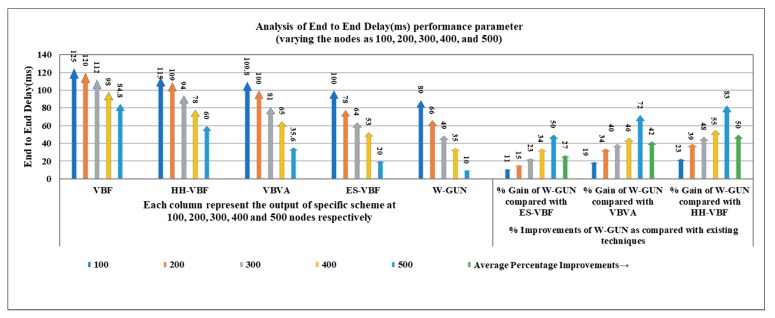
Detailed percentage-centric performance gain of W-GUN focusing on end-to-end delay.

A more detailed description of the end-to-end delay performance gain of the proposed framework is given in Table 1 with the comparative investigation of the frameworks HH-VBF, VBVA and ES-VBF, as described in the literature. It can be observed that the average performance gain of W-GUN in terms of percentage is 27%, 42%, and 50% for ES-VBF, VBVA and HH-VBF, respectively. This can be attributed to the fact that the natural underwater characteristics are not considered in the literature, which rather majorly relies on service quality and location-centric underwater relay nodes. However, W-GUN utilized underwater characteristics by the consideration of whales movement, resulting in a considerable performance gain. This performance gain is further represented in a more readable way in Figure 4, where percentage gain and end-to-end delay observations are shown to be in close relation. This verifies the results presented in Table 1 and Figure 3. Therefore, the proposed underwater framework outperforms the state-of-the-art techniques with considerably lower end-to-end delay.

**Figure 5:**
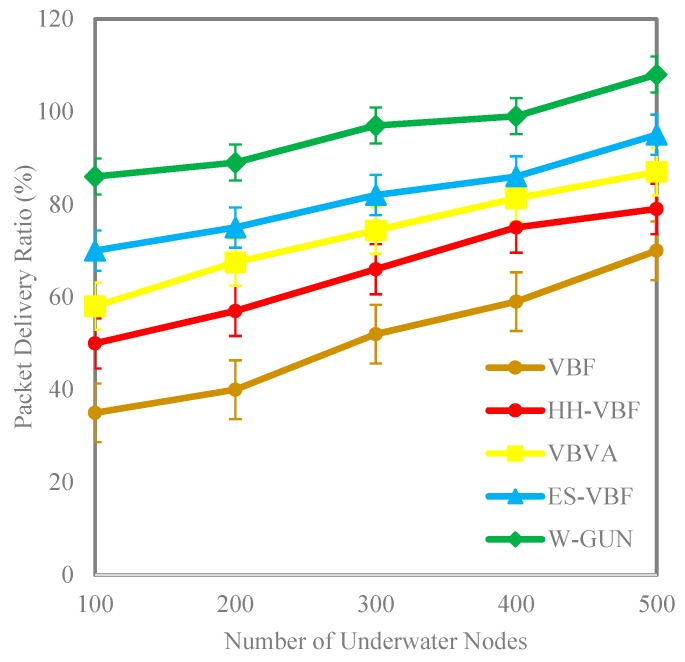
Packet delivery ratio versus the number of underwater nodes.

The results in Figure 5 show the comparison of packet delivery ratio performance between the proposed framework and state-of-the-art techniques with varying underwater network density in the range of 100–500 sensor nodes. It can be observed that the packet delivery ratio is significantly higher for the proposed underwater relay optimization framework compared to state-of-the-art techniques. The better packet delivery ratio for the proposed framework can be attributed to the utilization of underwater environmental characteristics in relay node optimization, ultimately paying as a higher packet delivery rate. Furthermore, an underwater environmental scenario-centric delivery path is the target of the proposal’s discovery mechanism, in order to have more stable nodes in the path for a higher packet delivery ratio. This means that the proposed underwater relay node optimization framework selects the more stable underwater routes with the optimal number of underwater relay nodes. Therefore, the delivery path consists of stable underwater relay nodes in our proposed approach. Thus, the packet delivery ratio is considerably higher compared with existing schemes in the underwater literature.

**Figure 6:**
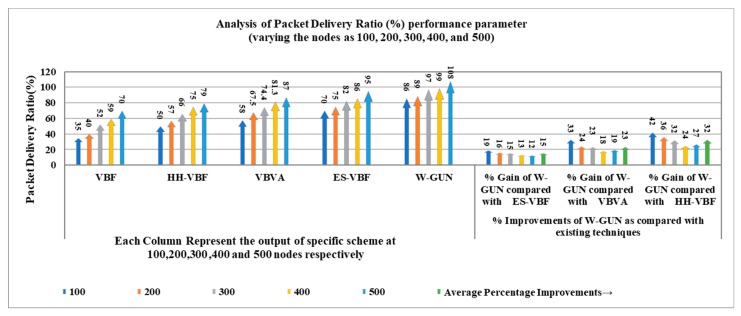
Detailed percentage-centric performance gain of W-GUN focusing packet delivery ratio.

A more detailed analysis of performance gain in terms of packet delivery ratio of the proposed framework is presented in Table 2. This comparative investigation considered the underwater state-of the-art literature for highlighting the respective performance gains against the framework in this study. It can be observed that the average performance gain of the proposal in terms of percentage is 15%, 23%, and 32% for ES-VBF, VBVA and HH-VBF, respectively. The reason behind this the utilization of underwater characteristics for identifying network dynamics which is not considered in the existing literature. The existing techniques have utilized service quality and location of underwater relay nodes for making data delivery decisions. However, the proposed framework considers underwater network dynamics resulting in significant performance gain in the packet delivery ratio. This performance gain is further shown in a more scientifically understandable way in Figure 6. Here, the percentage gain as well as packet delivery ratio observations are presented close together to make it easy to highlight the performance benefits of the proposal in comparison with the literature. This is also helpful in validating the results shown in Table 2 and Figure 5. Thus, the proposed underwater framework provides a better packet delivery ratio in underwater environments compared to state-of-the-art techniques.

**Figure 7:**
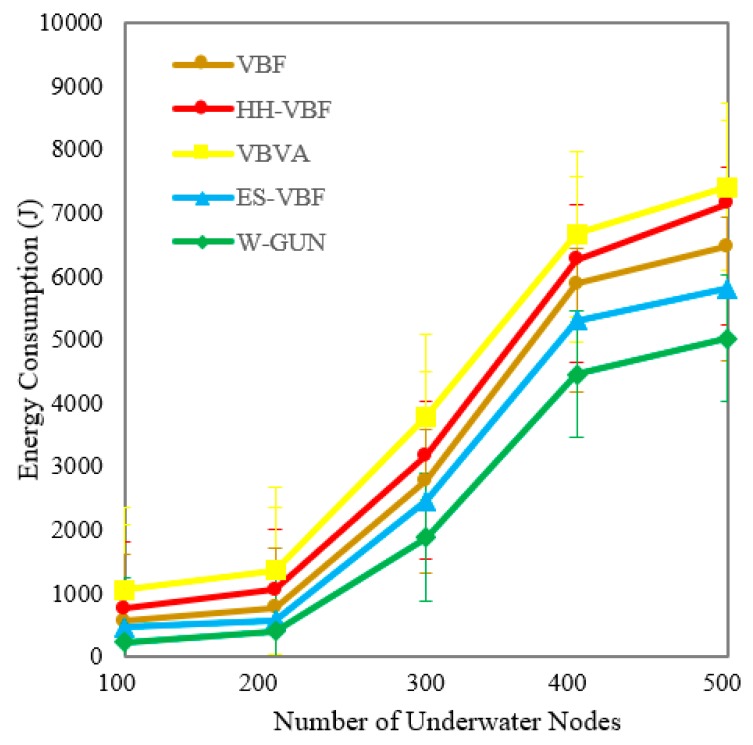
Energy consumption versus the number of underwater nodes.

A comparative analysis between the proposed framework and state-of-the-art techniques is presented in Figure 7 for energy consumption performance as a function of the number of underwater sensor nodes in the underwater network. It can be observed that the energy consumption is considerably lower for the proposed underwater relay optimization method W-GUN than the state-of-the-art techniques. It is evident that the optimal selection of underwater relay nodes reduces the energy consumption of the overall network, resulting in better utilization of the energy of underwater nodes. For lower energy consumption, the underwater routing path should be as optimal as possible. The proposed optimization framework selects the optimal number of underwater relay nodes for optimizing the energy usage at the node level. Therefore, the route consists of stable underwater nodes as well as the minimum number of underwater relay nodes in our proposed framework. In particular, it can be observed that energy consumption reaches more than 7000 j for VBVA with 500 sensor nodes in the underwater network. In the case of our proposal, it reaches up to approximately 5000 j with similar 500 sensor nodes underwater network density. Here, we want to clarify that the energy consumption is of the overall underwater network, considering all the sensor nodes’ energy consumption. As we have specified in our experimental setting description, total simulation time considered for each simulation experiment was 1500 s and each point considered in the result is an average 50 simulation runs. Essentially, after our simulation time, overall 14% of underwater network energy has been consumed in the case of VBVA and approximately 10% of underwater network energy has been consumed in the case of the proposed framework. Therefore, energy consumption is lower considerably compared with existing schemes in the underwater literature.

**Figure 8:**
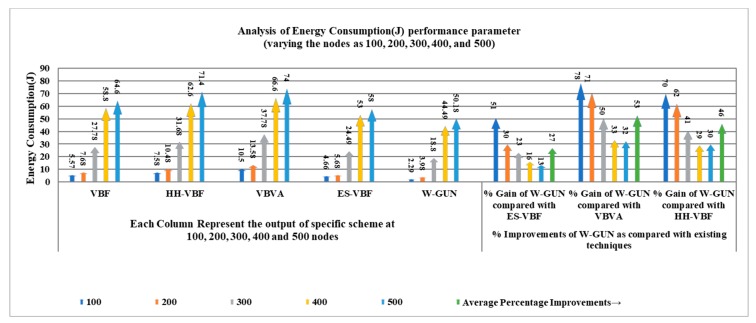
Detailed percentage-centric performance gain of W-GUN focusing energy consumption.

The overall energy consumption of the proposed framework is investigated in detail in Table 3 with a comparison to the literature, focusing on respective performance gain percentage. It can be noted that the average energy consumption performance gain of the proposed framework in percentage is 27%, 53%, and 46% for ES-VBF, VBVA and HH-VBF, respectively. This can be attributed to the fact that the optimal and stable underwater nodes are considered in the proposed framework, in comparison with the literature majorly relying on the quality of service and location-centric underwater relay nodes. W-GUN utilized underwater characteristics resulting in a considerable energy performance gain. This energy performance gain is further represented in a more understandable way in Figure 8, where percentage gain and energy consumption observations are shown in close relation for better clarity and understanding. This verifies the results presented in Table 3 and Figure 7. Therefore, the proposed underwater framework outperforms the state-of-the-art techniques with considerably lower energy consumption.

**Figure 9:**
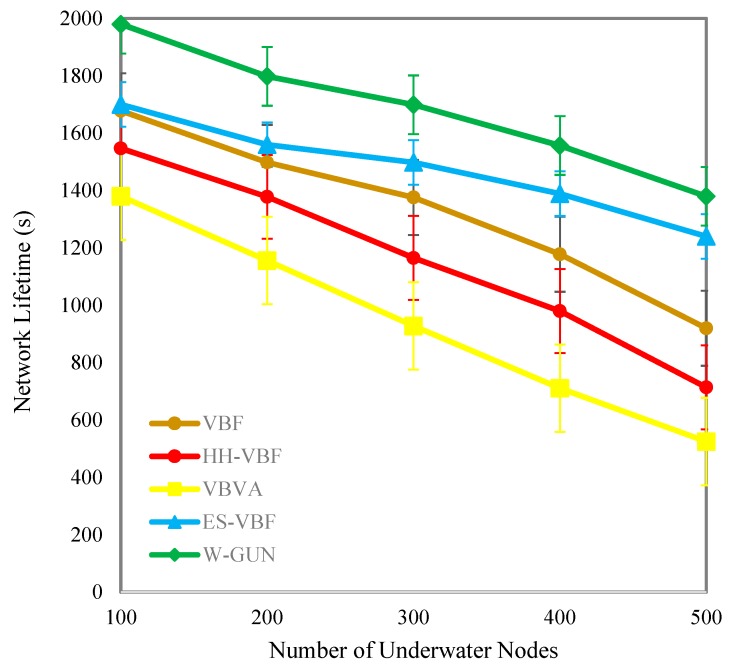
Network lifetime versus the number of underwater nodes.

The overall network lifetime as a function of the number of sensor nodes in the underwater network is comparatively investigated in Figure 9, considering the proposed framework and the state-of-the-art techniques. Similar network density in the range of 100–500 sensor nodes is considered for this experiment. It can be easily noticed that the network lifetime is effectively longer for the proposed underwater relay optimization framework than the compared state-of-the-art techniques. It is evident that the optimal selection of underwater relay nodes enhances the communication performance of the overall network in terms of a longer network lifetime. For a durable network lifetime, the underwater communication path should be optimal. The aim of the proposed optimization method is to select the maximal number of underwater communication routes with a minimal number of underwater relay nodes. Therefore, the route consists of the minimum number of underwater relay nodes in our proposed approach. Therefore, the network lifetime is longer considerably compared with existing schemes in the underwater literature.

**Figure 10:**
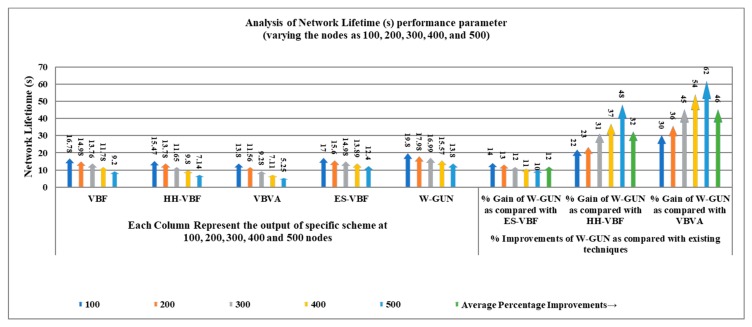
Detailed percentage-centric performance gain of W-GUN focusing network lifetime.

The longer network lifetime-centric performance benefits of the proposed framework are explored in detail in Table 4 in terms of percentage gain compared with the literature. The average performance gain of W-GUN in terms of network lifetime percentage can be notes as 12%, 32%, and 46% for literature including ES-VBF, HH-VBF and, VBVA respectively. The performance benefits can be reasoned to the fact that the natural underwater characteristics are not considered in the literature, which rather majorly relied on the quality of service and location information of underwater relay nodes. However, the proposed framework has utilized underwater characteristics, resulting in considerable performance gain and a longer network lifetime. This network lifetime performance gain is further represented in a more readable way in Figure 10. Here, the percentage gain and network lifetime observations are shown in close relation so that they can be analyzed relatively. This is also verifying the results presented in Table 4 and Figure 9. Therefore, the proposed underwater framework shows a longer network lifetime compared to the state-of-the-art techniques in the underwater literature.

**Figure 11:**
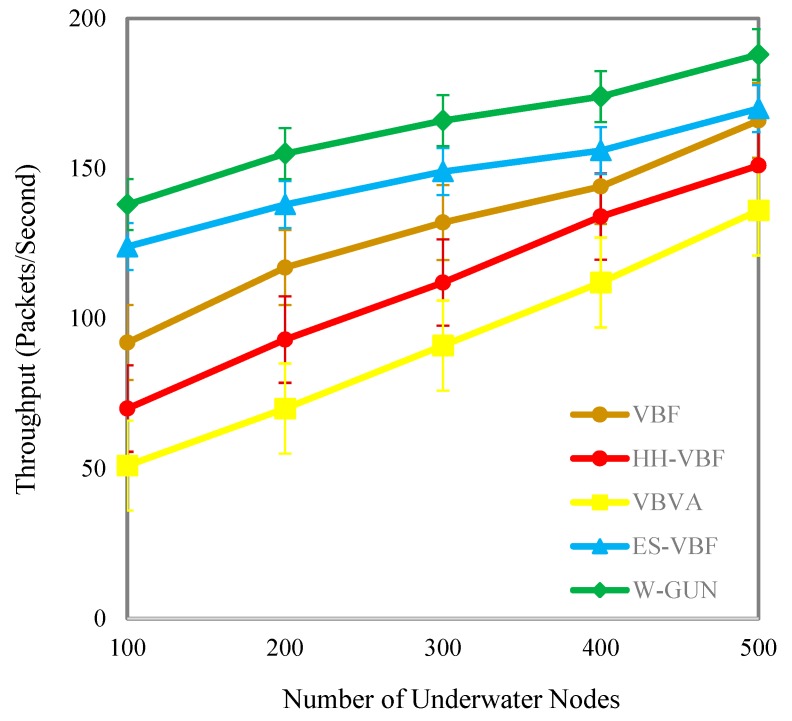
Throughput versus the number of underwater nodes.

The throughput performance of the proposed framework is comparatively studied in the results presented in Figure 11, with varying numbers of sensor nodes in the underwater network environment. The throughput of the proposed framework is significantly higher for the considered underwater relay optimization environment compared to state-of-the-art techniques. The better throughput of the proposal is due to the utilization of stable and lower delay-centric underwater relay nodes. In the framework, an underwater environmental scenario-centric routing path is discovered to have more stable nodes in the delivery path. In other words, the proposed underwater relay node optimization framework selects the more stable underwater routes compared to the considered existing literature with the optimal number of underwater relay nodes. The delivery path consists of stable underwater relay nodes in our proposed framework. Thus, the throughput performance of the proposal is considerably higher compared with existing schemes in the underwater literature.

**Figure 12:**
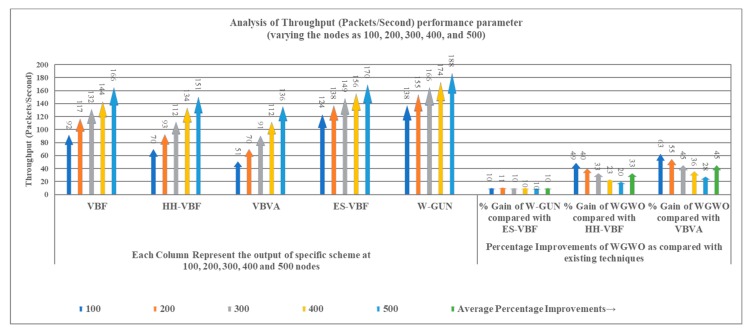
Detailed percentage-centric performance gain of W-GUN focusing throughput.

The benefits in terms of the throughput of the proposed framework are analyzed as performance gain in Table 5. It is a comparative investigation between the proposed framework and underwater state-of-the-art techniques. The average throughput performance gain of W-GUN in terms of percentage is 10%, 33%, and 45% for the underwater literature including ES-VBF, HH-VBF and, VBVA respectively. The reason behind the better throughput is the utilization of underwater characteristics for identifying network dynamics. However, W-GUN considers underwater network dynamics, resulting in significant performance gains in throughput. These performance benefits are further evident in Figure 12 in a more scientifically understandable way. Here, the percentage gain and throughput observations are presented in close relation so that they can be analyzed relatively. This is helpful in validating the results shown in Table 5 and Figure 11. Thus, the proposed underwater framework provides higher throughput in underwater environments compared to the state-of-the-art techniques.

## 5. Conclusions

In this paper, we have presented an underwater relay node optimization framework (W-GUN) for enabling green computing in underwater networking. The underwater relay nodes are selected to cover the maximal paths with the help of an adapted whale and wolf optimization algorithm. The position of underwater relay nodes is varied and checked for optimal deployment to reduce the number of underwater relay nodes for green underwater networking. The adapted W-GUN framework has been designed by integrating underwater-centric optimization techniques, namely a whale and wolf optimization algorithm. Moreover, the performance of the proposed framework is compared with the existing techniques ES-VBF, VBVA and HH-VBF. The proposed methodology is analyzed in terms of various underwater-centric metrics including end-to-end delay, packet delivery ratio, energy consumption, network lifetime, and throughput by varying the number of underwater sensor nodes in the network. The average percentage improvements reflect the effectiveness of the proposed scheme compared with the state-of-the-art underwater techniques. In addition, from the results attained, it can be concluded that the proposed optimal underwater relay selection scheme is effective in terms of improving the network lifetime with less energy consumption. In future works, the authors will focus on using deep learning-centric optimization in order to further evolve the understanding of underwater network environments in optimal relay node selection and route optimization based on network dynamics.

## Figures and Tables

**Table 1:** Descriptive performance observation of W-GUN in terms of end-to-end delay.

Delay (ms) Comparison with Varying Number of Nodes						% Improvements of W-GUN Compared with Existing Techniques		
Nodes	VBF	HH-VBF	VBVA	ES-VBF	W-GUN	% Gain of W-GUN Compared with ES-VBF	% Gain of W-GUN Compared with VBVA	% Gain of W-GUN Compared with HH-VBF
100	125	115	109.8	100	89	11	19	23
200	120	109	100	78	66	15	34	39
300	112	94	81	64	49	23	40	48
400	98	78	65	53	35	34	46	55
500	84.8	60	35.6	20	10	50	72	83
Average % Improvement →						27	42	50

**Table 2:** Descriptive performance observation of W-GUN in terms of packet delivery ratio.

Packet Delivery Ratio (%) Comparison with Varying Number of Nodes						% Improvements of W-GUN Compared with Existing Techniques		
Nodes	VBF	HH-VBF	VBVA	ES-VBF	W-GUN	% Gain of W-GUN Compared with ES-VBF	% Gain of W-GUN compared with VBVA	% Gain of W-GUN compared with HH-VBF
100	35	50	58	70	86	19	33	42
200	40	57	67.5	75	89	16	24	36
300	52	66	74.4	82	97	15	23	32
400	59	75	81.3	86	99	13	18	24
500	70	79	87	95	108	12	19	27
Average % Improvement →						15	23	32

**Table 3:** Descriptive performance observation of W-GUN in terms of energy consumption.

Energy Consumption (J) Comparison with a Varying Number of Nodes						% Improvement of W-GUN Compared with Existing Schemes		
Nodes	VBF	HH-VBF	VBVA	ES-VBF	W-GUN	% Gain of W-GUN Compared with ES-VBF	% Gain of W-GUN Compared with VBVA	% Gain of W-GUN Compared with HH-VBF
100	557	758	1050	466	229	51	78	70
200	768	1048	1358	568	398	30	71	62
300	2778	3168	3778	2449	1880	23	50	41
400	5880	6260	6660	5300	4449	16	33	29
500	6460	7140	7400	5800	5018	13	32	30
Average % Improvement →						27	53	46

**Table 4:** Descriptive performance observation of W-GUN in terms of network lifetime.

Network Lifetime (s) Comparison with Varying Number of Nodes						% Improvements of W-GUN Compared with Existing Techniques		
Nodes	VBF	HH-VBF	VBVA	ES-VBF	W-GUN	% Gain of W-GUN compared with ES-VBF	% Gain of W-GUN compared with HH-VBF	% Gain of W-GUN compared with VBVA
100	1678	1547	1380	1700	1980	14	22	30
200	1498	1378	1156	1560	1798	13	23	36
300	1376	1165	928	1498	1699	12	31	45
400	1178	980	711	1389	1557	11	37	54
500	920	714	525	1240	1380	10	48	62
Average % Improvement →						12	32	46

**Table 5:** Descriptive performance observation of W-GUN in terms of throughput.

Throughput (Packets/Second) Comparison with Varying Number of Nodes						Percentage Improvements of W-GUN Compared with Existing Techniques		
Nodes	VBF	HH-VBF	VBVA	ES-VBF	W-GUN	% Gain of W-GUN Compared with ES-VBF	% Gain of W-GUN Compared with HH-VBF	% Gain of W-GUN Compared with VBVA
100	92	70	51	124	138	10	49	63
200	117	93	70	138	155	11	40	55
300	132	112	91	149	166	10	33	45
400	144	134	112	156	174	10	23	36
500	166	151	136	170	188	10	20	28
Average % Improvement →						10	33	45
